# Hemophagocytic Lymphohistiocytosis Secondary to Obstructive Uropathy and Escherichia coli Bacteremia

**DOI:** 10.7759/cureus.41779

**Published:** 2023-07-12

**Authors:** Reese Hofstrand, Bradley Casey, Elizabeth Onyeaso

**Affiliations:** 1 Internal Medicine, Cape Fear Valley Medical Center, Fayetteville, USA; 2 Internal Medicine/Infectious Disease, Cape Fear Valley Medical Center, Fayetteville, USA

**Keywords:** escherichia coli, hyperbilirubinemia, severe sepsis, obstructive uropathy, hemophagocytic lymphohistiocytosis (hlh), e coli bacteremia

## Abstract

Hemophagocytic lymphohistiocytosis (HLH) is a rare syndrome in which widespread activation of the immune system causes multi-organ tissue damage. HLH is a serious and potentially fatal disorder characterized by excessive immune system activation. It is characterized by a dysregulation in natural killer (NK) T-cell function, causing activation of lymphocytes and histiocytes. These cells secrete a large number of inflammatory cytokines and infiltrate various tissues causing multi-organ system failure. The spectrum consists of hereditary or “primary” HLH that comprises genetically heterogeneous conditions, occurring during childhood. The secondary form presents later in life and is associated with several conditions mainly malignancy, autoimmune diseases, viral or bacterial infections, and hematological diseases. Here we present an interesting case in which a 39-year-old patient presented with a complaint of shortness of breath. He was diagnosed with obstructive uropathy in the emergency department and subsequently developed acute liver injury, acute kidney injury, *Escherichia coli* bacteremia, and was diagnosed with HLH with comorbid bacteremia.

## Introduction

Hemophagocytic lymphohistiocytosis (HLH) is a life-threatening disorder characterized by unbridled activation of cytotoxic T lymphocytes, natural killer (NK) cells, and macrophages resulting in hypercytokinemia and immune-mediated injury of multiple organ systems [1]. It is seen in both children and adults and is recognized as primary which is driven by underlying genetic mutations that abolish critical proteins required for normal function of cytotoxic T cells and NK cells. It can also be secondary to malignant, infectious, or autoimmune stimulus without an identifiable underlying genetic trigger [1]. This particular case was unusual due to the presentation with an uncommon infectious organism (*Escherichia Coli*) that is only occasionally mentioned in previous case reports. HLH is not usually linked with obstructive uropathy in the literature.

## Case presentation

**Figure 1:**
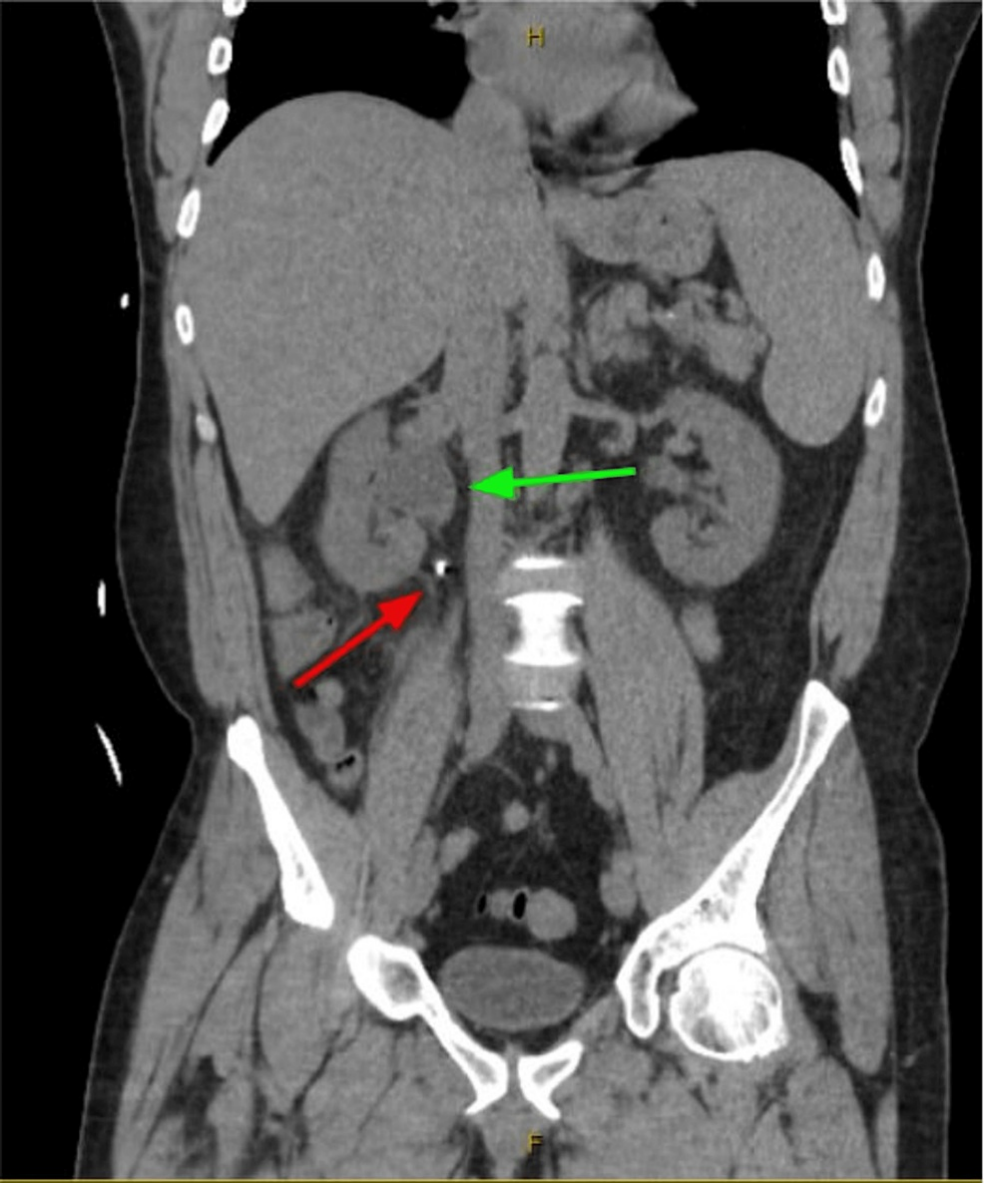
CT abdomen showing 7mm right ureteral stone (red arrow) with hydronephrosis (green arrow)

**Figure 2:**
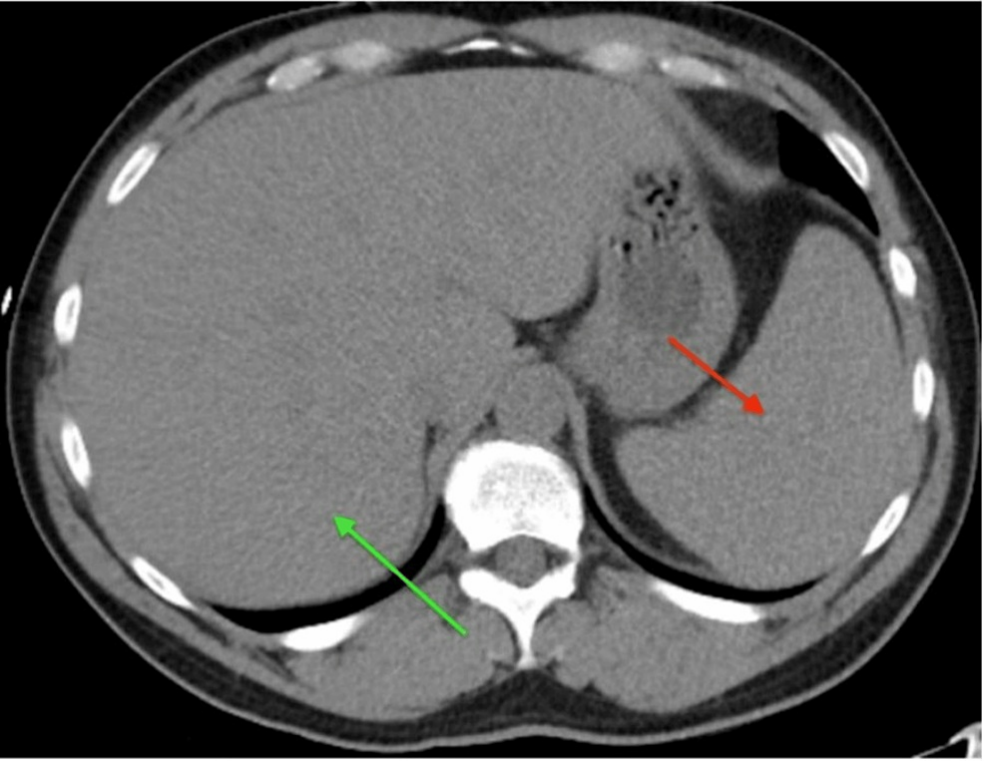
CT abdomen showing splenomegaly (red arrow) and liver with mild hepatomegaly (green arrow)

The patient is a 53-year-old male with no past medical history who was transferred from an outside facility with complaints of generalized weakness, nausea, and non-bilious/non-bloody vomiting for five months that has progressed over the past week. While at home, the patient recorded a maximum temperature of 103 °F. He stated that he had intermittent bouts of generalized weakness, fatigue, and muscle aches for the past five months after going on a hiking trip with his son and stepping into poison ivy. He reported that he went to his primary care provider and was prescribed antihistamines as well as a steroid pack. He completed the antihistamine and steroid prescription, but the rash from poison ivy did not dissipate. He consulted a dermatologist, who prescribed him a second regimen of antihistamines and a tapering dose of steroids. Over the past week, his symptoms progressed and he developed persistent generalized weakness, nausea, fever of 103 °F, and vomiting. Initial blood work and trends can be seen in Table 1. A CT scan of the patient's abdomen and pelvis, which was part of the work-up, revealed mild hepatomegaly, mild splenomegaly, and right-sided hydronephrosis caused by a 7 millimeter stone in the proximal right ureter (Figures 1, 2).

**Table 1:** HLH workup laboratory results HLH: hemophagocytic lymphohistiocytosis

Laboratory Tests	Reference Range	Patient Results
Gamma-glutamyl transferase	9-64 U/L	94 U/L
Ferritin	23.9-336.2 ng/ml	1230.2 ng/ml
IL-2 receptor alpha	223-710 U/ml	4866 U/mL
Hepatitis A anti IGM	Negative	Negative
Hepatitis B surface antigen	Negative	Negative
Hepatitis B core antibody IgM	Negative	Negative
Hepatitis C antibody	0.0-0.9 s/co ratio	0.2 s/co ratio
Hepatitis C antibody quantitative	Negative	Negative
Antimitochrondrial M2 antibody	0 -20 U	Less than 20 U
Actin (smooth muscle) antibody	0-19 U	6 U
Ceruloplasmin	16-31 mg/dL	34.1 mg/dL
Copper	69-132 ug/dL	150 ug/dL
Alpha 1-antitrypsin	101-187 mg/dL	248 mg/dL
Ebstein-Barr virus antibody viral capsid antigen IgM	0-35.9 U/ml	Less than 36 U/mL
*Mycoplasma pneumoniae* antibody IgM	0-769 U/ml	Less than 770 U/mL
*Ehrlichia chaffeensis* IgG titer	Less than 1:64	Less than 1:64
*Ehrlichia chaffeensis* IgM titer	Less than 1:20	Less than 1:20
Human granulocytic *Ehrlichia chaffeensis *IgG titer	Less than 1:64	Less than 1:64
Human granulocytic *Ehrlichia chaffeensis* IgM titer	Less than 1:20	Less than 1:20

Patient's blood cultures returned positive for *E. coli* that was sensitive to all antibiotics on susceptibility testing. At this time, the patient was started on pipercillin-tazobactam and later de-escalated to ceftriaxone. Urology was consulted and surgical intervention was planned for the patient. Due to the concerns of hyperbilirubinemia and elevated transaminases, gastroenterology was consulted. Ultrasound abdomen was ordered to evaluate the main portal vein as well as the splenic vein, both of which were patent with normal color Doppler evaluation. It also described a normal liver echotexture not consistent with hepatic steatosis or cirrhosis. The differential was broad given the non-specific presentation, including HIV, infectious hepatitis, budchiari syndrome, Wilson's disease, hemochromocytosis, erlichia, and autoimmune hepatitis. When the common diseases were ruled out, given the elevated ferritin HLH labs were sent and came back as positive several days later (Table 1).

Due to the patient's continuing to increase transaminases and hyperbilirubinemia gastroenterology recommended MRI of the abdomen. The liver at that time still demonstrated slight hepatomegaly without any liver masses, and the spleen demonstrated mild splenomegaly with no splenic masses. Despite multiple platelet transfusions, the patient's platelet count remained low. A smear was performed, which showed neutrophilia with cytoplasmic toxic granulation, but no significant left shift, schistocytes, or blasts. In addition, it showed thrombocytopenia without platelet clumps. With antibiotic treatment and supportive care over the course of five days, the patient's labs improved (Table 2).

**Table 2:** Trended laboratory results during hospitalization

Laboratory Tests	Reference Range	Day 1	Day 2	Day 3	Day 4	Day 5	Day 6	Day 7
Platelets	150-450 x 10*3/uL	60x 10*3/uL	14 x 10*3/uL	16x 10*3/uL	47x 10*3/uL	81x 10*3/uL	176x 10*3/uL	195x 10*3/uL
Creatinine	0.60-1.30 mg/dL	6.42 mg/dL	7.54 mg/dL	6.21 mg/dL	3.29 mg/dL	2.17 mg/dL	1.51 mg/dL	1.44 mg/dL
Total bilirubin	0.3-1.0 mg/dL	7.7 mg/dL	7.6 mg/dL	8.4 mg/dL	3.6 mg/dL	3.9 mg/dL	3.6 mg/dL	2.9 mg/dL
Aspartate aminotransferase	13-39 U/L	74 U/L	60 U/L	66 U/L	45 U/L	40 U/L	25U/L	28 U/L
Alanine aminotransferase	7-52 U/L	139 U/L	97 U/L	81 U/L	72 U/L	70 U/L	67 U/L	56 U/L
Alkaline phosphatase	30-105 U/L	342 U/L	165 U/L	165 U/L	129 U/L	128 U/L	137 U/L	144 U/L

Patient eventually returned back to baseline and was discharged. At outpatient follow-up six months later, the patient showed clinical improvement and his laboratory results returned to normal laboratory limits.

## Discussion

Diagnosing HLH can be challenging due to its similarities in presentation and laboratory results with other hematologic disorders. While there is no unified diagnostic criteria for HLH, the Hscore and HLH-2004 criteria are commonly used scoring systems. Hscore has been validated to assist in diagnosis of HLH based on clinical (immunosuppression, fever, organomegaly, biologic (serum oxaloacetic transaminase, fibrinogen levels, cytopenia, ferritin, triglyceride levels), and cytologic (hemophagocytosis features on bone marrow aspirate) [2]. HLH-2004 diagnostic criteria involve either the molecular diagnostic consistent with HLH or meeting at least five of eight criteria: fever, splenomegaly, cytopenias affecting two or more of three lineages, hypertriglyceridemia or low fibrinogen, hemophagocytosis in bone marrow, spleen, or lymph nodes, or sCD25 IL-2 2400 U/mL, low or no NK cell activity, Ferritin >500 micrograms per liter) [3]. Our patient was not treated with steroids due to long laboratory testing times and underlying sepsis. In this case, no bone marrow aspiration was performed, no fibrinogen test was conducted, and the triglyceride level from a previous encounter was used. Laboratory turnaround times were prolonged and the patient was treated for other concurrent conditions and the additional testing was unlikely to clinically benefit the patient. However, his high score of 224 points based on the tested variables gave a 96-98% probability of HLH and he did meet five criteria (Table 1) of the HLH-2004 criteria.

HLH is classified as either secondary HLH or primary HLH. Primary HLH involves Mendelian inherited conditions leading to HLH. Secondary HLH is most commonly triggered by viral infections such as influenza, cytomegalovirus, Epstein-Barr virus, HIV, bacterial infections, malignancy (most commonly lymphoma), and macrophage activation syndrome [3]. Infections have been the most commonly described trigger of secondary HLH [4]. Bacterial infections of all types represent 9% of reported HLH triggers [5]. Only one case of obstructive uropathy with subsequent *E. Coli* bacteremia leading to HLH has been reported [6]. HLH associated with Gram-negative bacteremia (*Actinobacter*) and *E. Coli* associated with necrotizing fasciitis has been described as well [7,8].

The treatment for HLH depends on the etiology. For non-infection-related HLH, the treatment is based on the HLH-94 protocol. HLH-specific therapy can achieve approximately a 50% survival rate. New evidence shows that treatment should reflect the heterogeneity of the etiologies of HLH, but newer protocols have yet to be developed. HLH is treated with eight weeks of etoposide and dexamethasone induction with a taper. Cyclosporine is added at week nine. However, infection-related protocols are slightly different and focus on the treatment of the underlying condition due to the risk of immunosuppression and decompensation in this population [1]. Treatment algorithms have been proposed for viral triggers but not for bacterial infections. In this case, the infection was treated without steroid and etoposide therapy with resolution of symptoms.

## Conclusions

HLH is an uncommon disease that arises from many different triggers. Evaluating HLH is challenging due to its clinical and laboratory similarities with other conditions, and treatment algorithms for infection-related presentations are still being investigated. This case highlights a rare presentation of HLH associated with obstructive uropathy and *E. coli* bacteremia, a scenario that is rarely described in the literature.
